# Genetic and Epigenetic Changes in Chromosomally Stable and Unstable Progeny of Irradiated Cells

**DOI:** 10.1371/journal.pone.0107722

**Published:** 2014-09-24

**Authors:** Janet E. Baulch, Umut Aypar, Katrina M. Waters, Austin J. Yang, William F. Morgan

**Affiliations:** 1 Department of Radiation Oncology, University of California Irvine, Irvine, California, United States of America; 2 Department of Laboratory Medicine and Pathology, Mayo Clinic, Rochester, Minnesota, United States of America; 3 Fundamental and Computational Sciences Directorate, Pacific Northwest National Laboratory, Richland, Washington, United States of America; 4 Department of Anatomy and Neurobiology, University of Maryland School of Medicine, Baltimore, Maryland, United States of America; 5 Biological Sciences Division, Pacific Northwest National Laboratory, Richland, Washington, United States of America; University of Tokyo, Japan

## Abstract

Radiation induced genomic instability is a well-studied phenomenon, the underlying mechanisms of which are poorly understood. Persistent oxidative stress, mitochondrial dysfunction, elevated cytokine levels and epigenetic changes are among the mechanisms invoked in the perpetuation of the phenotype. To determine whether epigenetic aberrations affect genomic instability we measured DNA methylation, mRNA and microRNA (miR) levels in well characterized chromosomally stable and unstable clonally expanded single cell survivors of irradiation. While no changes in DNA methylation were observed for the gene promoters evaluated, increased LINE-1 methylation was observed for two unstable clones (LS12 and CS9) and decreased Alu element methylation was observed for the other two unstable clones (115 and Fe5.0–8). These relationships also manifested for mRNA and miR expression. mRNA identified for the LS12 and CS9 clones were most similar to each other (261 mRNA), while the 115 and Fe5.0–8 clones were more similar to each other, and surprisingly also similar to the two stable clones, 114 and 118 (286 mRNA among these four clones). Pathway analysis showed enrichment for pathways involved in mitochondrial function and cellular redox, themes routinely invoked in genomic instability. The commonalities between the two subgroups of clones were also observed for miR. The number of miR for which anti-correlated mRNA were identified suggests that these miR exert functional effects in each clone. The results demonstrate significant genetic and epigenetic changes in unstable cells, but similar changes are almost as equally common in chromosomally stable cells. Possible conclusions might be that the chromosomally stable clones have some other form of instability, or that some of the observed changes represent a sort of radiation signature and that other changes are related to genomic instability. Irrespective, these findings again suggest that a spectrum of changes both drive genomic instability and permit unstable cells to persist and proliferate.

## Introduction

Radiation induced genomic instability is a delayed, persistent effect of ionizing radiation exposure that manifests in the unirradiated progeny of irradiated cells as an increased frequency of mitotically heritable genetic alterations. Radiation induced genomic instability is a non-targeted phenomenon that is thought to contribute to radiation carcinogenesis, however the mechanisms underlying this process are poorly understood [1], [2]. The spectrum of alterations observed in cells exhibiting genomic instability include DNA double strand breaks (DSBs), mutations, changes in gene expression, disruption of mitochondrial processes, chromosomal rearrangements, cell cycle arrest, and apoptotic cell death. Studies from a number of laboratories have attempted to elucidate the mechanisms that underlie the initiation and/or perpetuation of genomic instability [3]–[7]. Based on such studies, many different mechanisms have been invoked, including persistent oxidative stress, mitochondrial dysfunction, increased cytokine secretion, and epigenetics [8]–[12]. However, none of these mechanisms alone seem to be sufficient to induce genomic instability, suggesting that radiation induced genomic instability is a multifactorial phenomenon.

Epigenetic mechanisms include altered DNA methylation, histone and chromatin modifications, and microRNA (miR) all of which can affect gene expression and cellular phenotype. Epigenetic aberrations have been observed following irradiation and also play a role in carcinogenic processes [3]–[5]. In cancer cells, global hypomethylation can lead to the initiation of genomic instability [13]. In particular hypomethylation of repeat elements, including long interspersed nuclear elements 1 (LINE-1) and Alu elements, can lead to chromosomal instability, translocations, and gene disruption caused by the reactivation of transposable DNA sequences [14]. In addition, transcriptional silencing of tumor suppressor genes can occur due to promoter hypermethylation and oncogene activation can occur due to promoter hypomethylation. MiR expression also plays an important role in the regulation of cellular pathways including cell proliferation, differentiation, and apoptosis by modulating gene expression [15]. Deregulation of miR expression can result in disruption of these cellular pathways, contributing to carcinogenesis. Certain miR such as miR-34c, have also been shown to be involved in the control of genomic instability [16]. Similarly, changes to histone marks and chromatin conformation can aberrantly alter gene expression and cellular phenotype and are associated with carcinogenesis [17]. To date, studies have predominantly evaluated the direct epigenetic effects of irradiation and while little is known regarding the possible delayed epigenetic aberrations in the genomically unstable progeny of irradiated cells such changes are likely to contribute radiation induced genomic instability [3]–[5].

We hypothesize that epigenetic aberrations are perpetuated in chromosomally unstable cells exhibiting genomic instability and that these epigenetic aberrations play a mechanistic role in the unstable phenotype. To test this hypothesis, DNA methylation, mRNA and miR levels were measured in well characterized clonally expanded single cell survivors of either low linear energy transfer (LET) X-irradiation or high LET iron (Fe) ion irradiation [18], [19] to evaluate possible correlations between altered epigenetic profiles and genome instability. The results demonstrate correlations between epigenetic changes and a cell exhibiting radiation induced genomic instability. In some instances these changes are likely to contribute to the unstable phenotype. However, similar to the other mechanisms that have been invoked to underlie the persistent instability phenotype, the genetic and epigenetic changes and differences in mRNA levels that we have identified in this study are unlikely to be sufficient to be the sole driver of radiation induced genomic instability.

## Materials and Methods

Unless otherwise noted, reagents were obtained from Sigma (St. Louis, MO). All primer sequences and annealing temperatures are listed in Table S1. Primers were obtained from Integrated DNA Technology, San Diego CA.

### Cell Culture and Analysis of Chromosome Stability

The human-Chinese hamster ovary (CHO) hybrid cell line, GM10115, contains a single copy of human chromosome 4 in a background of 20–24 hamster chromosomes (Human Genetic Mutant Cell Repository, Camden, NJ). The parental GM10115 cell line and the GM10115-derived stable clones (114, 118) and unstable clones (CS9, LS12, 115 and Fe5.0–8) were maintained in high glucose Dulbecco's Modified Eagle Medium (DMEM) containing 10% fetal bovine serum (FBS, Hyclone, Logan, UT), 2 mM L-glutamine (Invitrogen, Carlsbad, CA), and 0.2 mM L-proline at 34°C with 5% CO_2_. Cell cultures were routinely screened to exclude the presence of mycoplasma (Bionique Testing Laboratories, Inc., Saranac Lake, NY).

These stable and unstable cell lines were established and originally characterized by Limoli and colleagues following irradiations that took place in 1997 in the case of the CS9, LS12, 115, 114, and 118 clones [18], and in 2000 in the case of the Fe5.0–8 clone [19] The GM10115 cell line has a single copy of human chromosome 4 and this serves as the target for analysis of radiation induced instability. Following irradiation individual cells were clonally expanded then analyzed for rearrangements involving human chromosome 4 with the hamster chromosomes using fluorescence *in situ* hybridization (FISH) with chromosome 4 as the probe. A clone was classified as unstable if FISH analysis identified >3 clones showing unique rearrangements of chromosome 4 that make up >5% of the 200 metaphase cells analyzed. In this study each clone was re-characterized cytogenetically to confirm the stable/unstable nature of the clone as described previously [20]. A summary of the instability status of the clones used in this study is given in Table 1.

**Table 1:** Cytogenetic classification of isogenic clonally expanded cell lines.

Clone ID	Radiation Exposure	Cytogenetic Classification	Reference
GM10115	Unirradiated Control	Parental	
114	10 Gy X-rays	Stable	[18]
118	10 Gy X-rays	Stable	[18]
CS9	10 Gy X-rays	Unstable	[18]
LS12	10 Gy X-rays	Unstable	[18]
115	10 Gy X-rays	Unstable	[18]
Fe5.0–8	5 Gy Fe ions	Unstable	[19]

### DNA Isolation and Bisulfite Treatment

DNA was extracted from all experimental groups in a single batch using the DNeasy kit using standard methods (Qiagen, Valencia, CA) and stored in TE buffer at −20°C. Bisulfite modification of genomic DNA was also performed in a single batch using the EpiTect bisulfite kit using standard methods (Qiagen). Bisulfite treated DNA was used in specific locus, bisulfite sequencing and repeat element methylation analyses.

### Specific Locus Methylation

Promoter DNA methylation for the *nuclear factor-kappa B*, *tumor suppressor in lung cancer 1* and *E-cadherin* (*NFκB*, *TSLC1*, and *CDH1*, respectively) was evaluated by methylation-specific PCR (MSP) as described previously [21]. Primers were designed based on human or mouse sequences, because hamster sequences were unavailable (Integrated DNA Technology, San Diego CA). Primers were tested for specificity to bisulfite treated DNA and no amplification of genomic DNA occurred. Positive and negative control reactions were performed for all PCR. Two biological replicate experiments were performed for each clone and locus. PCR products were resolved on 3% agarose gels, stained using ethidium bromide and images were digitally captured.

Promoter methylation of *NFκB* was also measured by bisulfite sequencing. Primers were tested for specificity to bisulfite treated DNA and no amplification of genomic DNA occurred. Sequencing was performed at the Institute for Genome Sciences, University of Maryland Biopark. Three biological replicates were performed.

### *Human NFκB* Genomic DNA Validation and mRNA levels

Primers for genomic *NFκB* sequence were used to determine presence of the genomic sequence. PCR products were resolved on 3% agarose gels, stained using ethidium bromide and images were digitally captured. Two biological replicate experiments were performed for each clone.

Total RNA was extracted from all experimental groups in a single batch using the miRNeasy Mini Kit (Qiagen) following standard kit procedures with a DNAse treatment to eliminate DNA contamination. RNA concentrations and purities were measured using a NanoDrop ND-1000 spectrophotometer (NanoDrop Technologies, Wilmington, DE). For all RNA, the 260/280 ratios were between 1.97–2.08, and the 260–320 ratios were above 1.5. cDNA was prepared from 1 µg RNA using 5× iScript Reaction Mix kit (Bio-Rad, Hercules CA). Negative control reactions omitting reverse transcriptase were included with all experiments. Quantitative PCR was performed using SsoFast EvaGreen Supermix (Bio-Rad). The efficiency of real-time PCR for each primer was calculated by analyzing a template dilution series, plotting the C_T_ values against the log template amount, and determining the slope of the resulting standard curve [22]. From the slope (S), efficiency was calculated using the following formula: PCR efficiency (%)  =  (10(−1/S) – 1) ×100. cDNA samples were assayed using a CFX 96 Real Time PCR detection system and CFX Manager software (Bio-Rad). The melting curve was determined at the end of the amplification by increasing the temperature from 65° C to 95° C with 0.5° C every 5 sec. Gene expression levels (Sp) for each individual sample were normalized relative to two housekeeping genes (HK) β-Actin and porphobilinogen deaminase (PBGD) and change in expression was evaluated using comparative 2^−(ΔCt)^ calculations. Three biological replicate experiments were performed for each clone.

### Repeat Element Methylation

LINE-1 and Alu repeat element DNA methylation was evaluated by combined bisulfite restriction analysis (COBRA) [23]. The details of this assay including PCR conditions and primer sequences have been published previously [21]. Four biological replicate experiments were performed for each clone and repeat element.

### Global DNA Methylation

Global DNA methylation was evaluated by methylation-sensitive arbitrarily primed PCR (MSAP-PCR) using a single primer, MLG2 [24]. The details of this assay including PCR conditions and the primer sequences have been published previously [21]. Four biological replicate experiments were performed for each clone.

The bands observed on the MSAP-PCR gels were excised and the DNA fragments purified using the MinElute Gel Extraction Kit (Qiagen). These PCR products were sequenced at the Biopolymer-Genomics Core (University of Maryland School of Medicine, Baltimore, MD) using the MLG2 primer. The sequences were identified using National Center for Biotechnology Information (NCBI) blasts. Bisulfite sequencing was used to validate the methylation status of identified sequences. PCR products were resolved on 3% agarose gels to confirm reaction quality, stained using ethidium bromide, and images were digitally captured. PCR products were cloned into competent bacteria using the TOPO cloning kit (Invitrogen) and subsequent plasmid mini preparation (Invitrogen). Sequencing was performed at the Biopolymer-Genomics Core using the T3 primer (5′-ATT AAC CCT CAC TAA AGG GA-3′). For each sample, ten sub-clones were sequenced from each of the clones used in this study.

### MiR Expression Array and Target Prediction

MiR microarray analysis of total RNA was performed by LC Sciences LLC (Houston, TX). Quality control for the integrity of total RNA, enrichment of miR from total RNA, labeling, hybridization to µParaflo microfluidics chip and scanning were performed using miRHuman/Mouse/Chinese Hamster miR array chips, based on Sanger miR-Base Release 12.0. MiR identified as differentially regulated for each clone as compared to the parental GM10115 cell line are provided in the miR Excel Workbook S1 (miR.xlsx). MiR target prediction was performed using three different computational programs, TargetScan [25], MicroRNA.org [26], MicroCosm (miRBase) [27]–[29]. Gene targets for which there was a consensus among the three databases were considered potential targets. Two replicate arrays were performed.

### mRNA Expression Arrays and Target Prediction

Gene expression microarray analysis was performed by LC Sciences using the Affymetrix GeneChip Mouse Genome 430 2.0 array. The mouse array was used because a hamster genome array was not available, and mouse was deemed the closest match. Quality control for the integrity of total RNA was performed and then the Affymetrix's GeneChip IVT Express kit was used for cDNA synthesis and *in vitro* transcription. mRNA identified as differentially regulated for each clone as compared to the parental GM10115 cell line are provided in the mRNA Excel Workbook S2 (mRNA.xlsx). mRNA that were differentially expressed were analyzed using the Database for Annotation, Visualization and Integrated Discovery (DAVID) [30], [31] pathway analysis based on gene ontology GOTERM BP 3, 4, and 5. Pathways were identified using the Functional Annotation Clustering feature (high classification stringency). Three replicate arrays were performed.

### Merging of miR and mRNA Data

To determine gene expression pathways affected by altered miR expression in clones exhibiting genomic instability, predicted gene targets of miR were merged with mRNA candidates using the Bioinformatics Resource Manager program version 2.1 [32], [33] and overlapping targets in all databases were subject to DAVID pathway analysis [30], [31]. Pathways, for which miR were up-regulated and target gene expression was down-regulated, or vice versa, were identified using the Functional Annotation Clustering feature (high classification stringency).

Candidate miR with an intensity signal ≥500 and involved in pathways of interest were validated using qRT-PCR. Total RNA used for the microarray analysis was reverse transcribed to cDNA template and TaqMan miR assays were performed in triplicate by LC Sciences. The hsa-miR-16 miR, which was expressed equally in all the samples on the microarray, was used as an internal control.

### Statistical Analyses

Unless otherwise noted above, means and standard errors were calculated for all data points from either 3 or 4 replicate experiments. The means were compared between samples by Student's t-test analysis using the StatPlus Mac software package (AnalystSoft, Vancouver, BC) and *P* values <0.05 were considered statistically significant. For the miR expression arrays, preliminary statistical analyses were performed on raw data normalized by the Locally-Weighted Regression (LOWESS) method on the background-subtracted data. ANOVA were then performed to identify differences in miR expression. Two replicate arrays were performed, so the significance threshold was set at *P*<0.10. For the mRNA expression arrays, three replicate arrays were performed and the data were normalized using the Robust multiarray analysis, and differentially regulated genes were identified with multiple testing and false discovery rate statistics at *P*<0.05 using the GeneSpring GX software (Agilent Technologies, Santa Clara, CA, USA).

## Results

### *NFκB* DNA Methylation and Gene Expression

**Figure 1:**
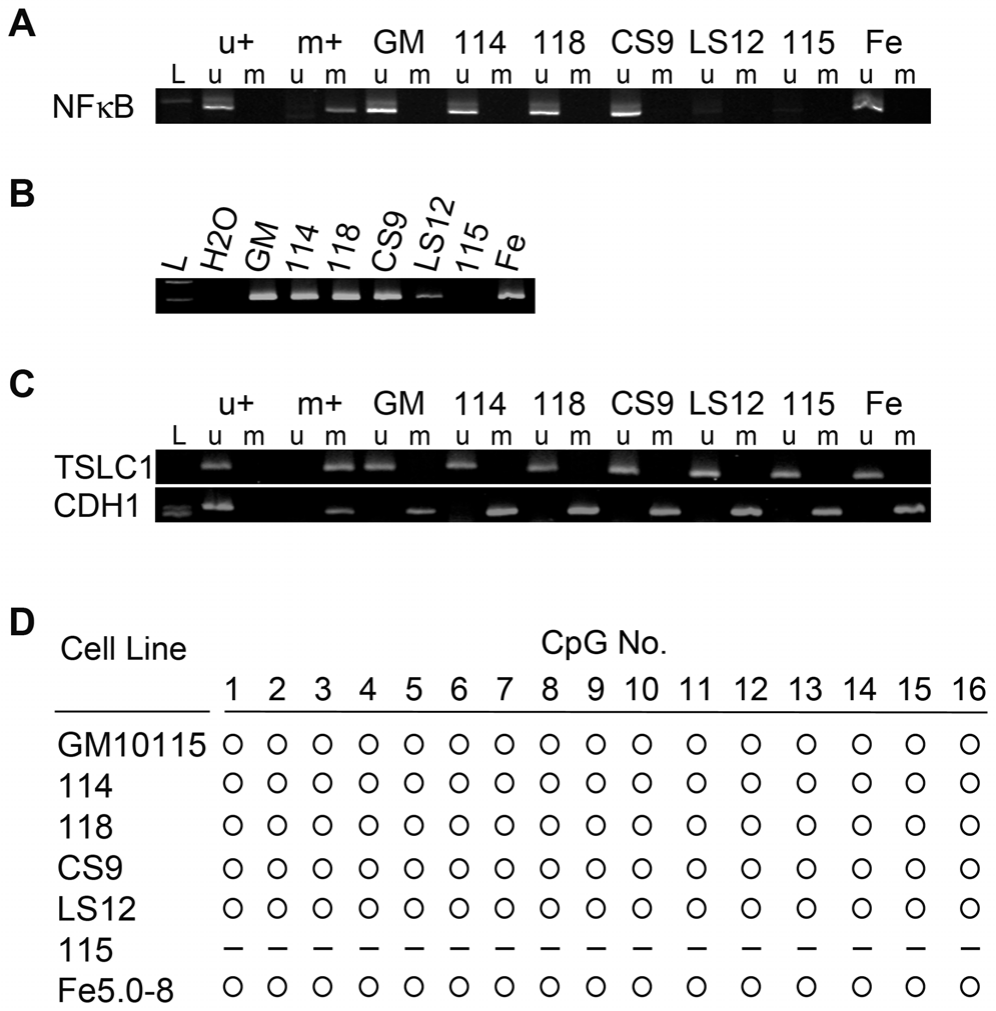
Human *NFκB* methylation status. Representative gels for A) methylation sensitive PCR of bisulfite modified DNA for the *NFκB* promoter; B) PCR of unmodified genomic DNA; and C) methylation sensitive PCR of bisulfite modified DNA for *TSLC1* and *CDH1* promoter methylation. D) Bisulfite sequencing for *NFκB* promoter. ‘L’ lanes indicate molecular weight ladders, ‘u’ lanes indicate PCR using primers specific to unmethylated promoter sequences; ‘m’ lanes indicate PCR using primers specific to methylated promoter sequences; ‘u+’ and ‘m+’ indicate respective positive control PCRs; the H2O lane indicates a PCR control containing no DNA template; open circles indicate unmethylated CpG; dashes indicate that no data was obtained).

Previous studies characterizing these clones implicated *NFκB* in the unstable phenotype [34]. In order to determine whether altered DNA methylation or expression of the *NFκB* gene plays a role in genomic instability, a promoter region of *NFκB* containing 4 CpG was evaluated using methylation sensitive PCR for comparison of the clones to the parental GM10115 cell line (Figure 1A). *NFκB* DNA methylation for the two stable clones and unstable clones CS9 and Fe5.0–8 matched that of the parental GM10115 cells. However, for the unstable clones LS12 and 115 no significant amplification was observed in either the unmethylated or the methylated *NFκB* PCR. To confirm this observation, PCR of the same sequence was performed using genomic DNA and sequence-specific primers rather than bisulfite converted DNA and methylation sensitive primers (Figure 1B). Low level amplification was observed for the LS12 clone, but none was observed for 115. For the LS12 cell line these results could suggest mono-allelic deletion of this region or that a subpopulation of cells within the LS12 cell line have bi-allelic loss of the region. For the 115 cell line these results could indicate bi-allelic loss. Additional PCRs using other primers demonstrated that the deletion extends from an undefined location in the promoter region, or upstream of it, downstream to exon 3 of the gene (data not shown). Analysis of the human *NFκB* sequence in the Genetic Information Research Institute (GIRI) database indicates the presence of a transposable element immediately upstream of the original forward primer's binding site, suggesting a possible hot spot for chromosomal breaks and rearrangement. Analysis of DNA methylation status for two other gene promoters, *TSLC1* and *CDH1*, showed no effects of the instability phenotype or history of irradiation on DNA methylation (Figure 1C).

Evaluating the CHO genome in general is somewhat complicated due to the unavailability of many hamster DNA sequences. As a result primers for our study were designed based on human or mouse sequences. Evaluating the *NFκB* gene in particular is more complicated for GM10115-derived cells because the cell line contains human chromosome 4 which is, coincidentally, the location of the human *NFκB* gene [35]. Consequently GM10115 cells possess both CHO and human *NFκB* genes. It was not clear whether our PCRs were amplifying human only or both human and CHO sequences. Bisulfite sequencing of *NFκB* was performed in an effort to obtain a more informative measure of the DNA methylation at the locus. No differences in DNA methylation were observed at any of the 16 CpG islands were evaluated for any of the clones (with the exception of the 115 clone, for which no data was obtained since the sequence did not amplify; Figure 1D). One hundred percent consensus was observed for all sequences, suggesting that only the human gene was being amplified in our genomic DNA and bisulfite treated DNA PCRs or that there was 100% homology between the human and CHO sequences.

**Figure 2:**
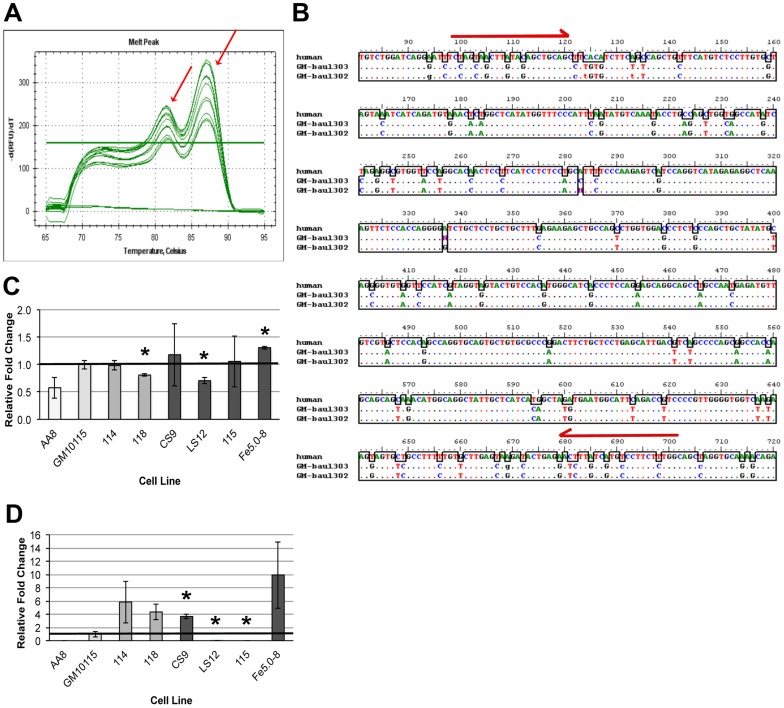
*NFκB* gene expression. A) Melt curve for *NFκB* qRT-PCR with the two PCR products indicated by red arrows. B) Sequence mismatch between human and CHO PCR products. Red arrows indicate species specific primer sites. C) CHO and D) human *NFkB* gene expression relative to the parental GM10115 cell line. Columns represent mean + SE for three experiments; * *P*<0.05, 2-tailed t-test.

Subsequently, qRT-PCR primers based on the human *NFκB* sequence were designed to evaluate gene expression and determine whether all clones expressed *NFκB* at levels similar to the parental cell line. Unlike the PCR for DNA, evaluation of qRT-PCR melt curves suggested that the RT-PCR primers were amplifying two unique PCR products (Figure 2A). Sequencing of these PCR amplicons demonstrated that one sequence had 100% homology to the predicted human sequence and the other had numerous mismatches (Figure 2B). The former sequence was not amplified in AA8 hamster cells that do not contain human chromosome 4 and it was not amplified in the 115 clone that had shown no amplification in the DNA methylation analyses. These findings indicate that GM10115-derived cells express both human and CHO *NFκB.* Based on these sequence data, species specific primers nested within the first qRT-PCR amplicon were designed for analysis of gene expression as indicated by the red arrows in Figure 2B. For CHO *NFκB*, the unstable LS12 clone and the stable 118 clone had subtle, but significantly decreased expression and the unstable Fe5.0–8 clone had significantly increased expression relative to the parental cell line (*P* = 0.03, 0.01, 0.007, respectively; Figure 2C). For human *NFκB* the unstable LS12 and 115 clones had undetectable levels of expression, correlating with the proposed deletion event (*P* = 0.0007; Figure 2D, Figure 1A). All other unstable and stable clones overexpressed human *NFkB* relative to the parental cell line with fold changes larger than those observed for CHO gene expression. However, these fold changes were significant only for the CS9 unstable clone (*P* = 0.02).

### Repeat Element and Global DNA methylation

**Figure 3:**
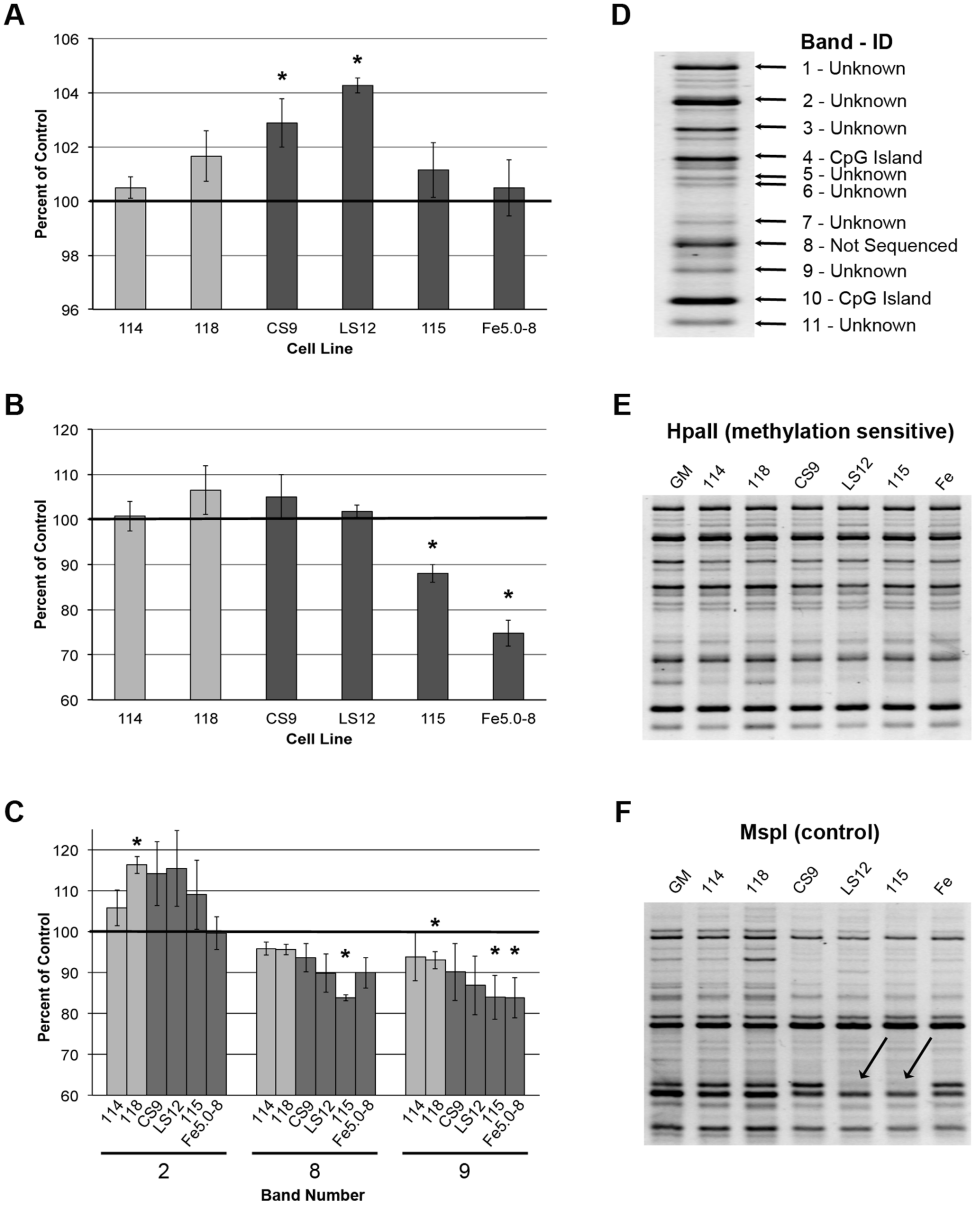
DNA methylation for stable and unstable clones normalized to the parental GM10115 cell line. A) LINE-1, B) Alu element, and C) global DNA methylation relative to the parental GM10115 cell line. D) 11 bands were analyzed for global DNA methylation and sequencing was able to provide identity for 2 of the amplicons. E) Representative gel for methylation sensitive HpaII digest PCR. F) Representative control (MspI) gel supports the hypothesis for possible deletion events in the LS12 and 115 cell lines. Arrows indicate missing band. In all cases the data represent mean +SE for four replicate experiments, * *P*<0.05, 2-tailed t-test.

Both repeat element and global DNA hypomethylation have been linked to genomic instability [36], [37]. For this reason, LINE-1 and Alu repeat element DNA methylation was evaluated by COBRA. For LINE-1, hypermethylation was observed in the unstable clones CS9 and LS12 relative to parental GM10115 cells (*P* = 0.02 and 0.01, respectively; Figure 3A). No change in methylation was observed for unstable clones 115 and Fe5.0–8 or for either of the stable clones. Hypomethylation of Alu elements was observed for the unstable clones 115 and Fe5.0–8 relative to parental (*P* = 0.03 and 0.005, respectively; Figure 3B). There was no change in Alu methylation for unstable clones CS9 and LS12 or for either of the stable clones.

Global DNA methylation was evaluated by MSAP-PCR. Genomic DNA was digested using the HpaII restriction enzyme that is sensitive to methylation at the internal cytosine of the recognition site, 5′…C^▵^CGG…3′. The enzyme will not cut if the internal cytosine is methylated. Since the MGL2 PCR primer binds at that cleavage site, presence or increase of a particular PCR product correlates CpG methylation. Changes in DNA methylation at this internal CpG were observed in one or more clones for three out of the eleven bands evaluated (Figure 3C). Band #2 was significantly hypermethylated in the stable clone 118 (*P* = 0.01). Band #8 was hypomethylated in the stable clone 118 and the unstable clone 115 (*P* = 0.04 and 0.001, respectively). Band #9 was hypomethylated in the stable clone 118 and unstable clones 115 and Fe5.0–8 (*P* = 0.04, 0.05, and 0.04, respectively).

The eleven PCR amplicons for which intensity was quantified were also isolated and sequenced (Figure 3D). Bands #4 and #10 were identified as CpG islands and the other nine bands were unidentified sequences. MSAP-PCR analysis indicated that DNA methylation for the two identified CpG island sequences was not different for any of the clones relative to control. Using primers specific to each of the CpG islands and bisulfite sequencing, this negative result was confirmed (data not shown). The banding pattern on the gel for the methylation sensitive HpaII enzyme digest and for the MspI control digests are shown in Figures 3E and 3F. The MspI gel prominently shows a missing amplicon in the LS12 and 115 samples as noted by arrows, correlating with the observation made in the *NFκB* experiments regarding the potential for deletion events in those clones (Figure 3F).

### mRNA Array Analyses

**Figure 4:**
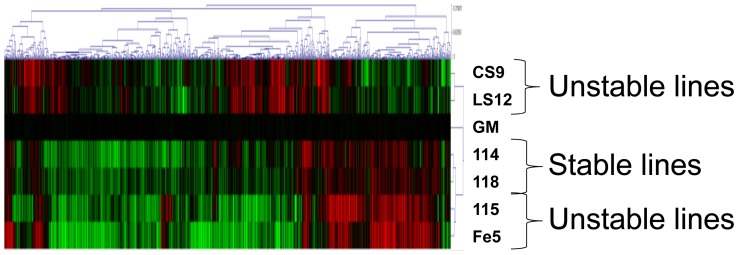
Representative heat map emphasizing the relationship among the various clones for significant changes in mRNA levels. For the purpose of this qualitative illustration, we present the heat map for the statistical threshold of *P*<0.10 where significant increases are red, significant decreases are green and no change is black. The actual numerical data used in the study were normalized using the Robust multiarray analysis, and differentially regulated genes were identified with multiple testing and false discovery rate statistics at *P*<0.05. Three replicate arrays were performed and the significance threshold was set at *P*<0.05.

**Figure 5:**
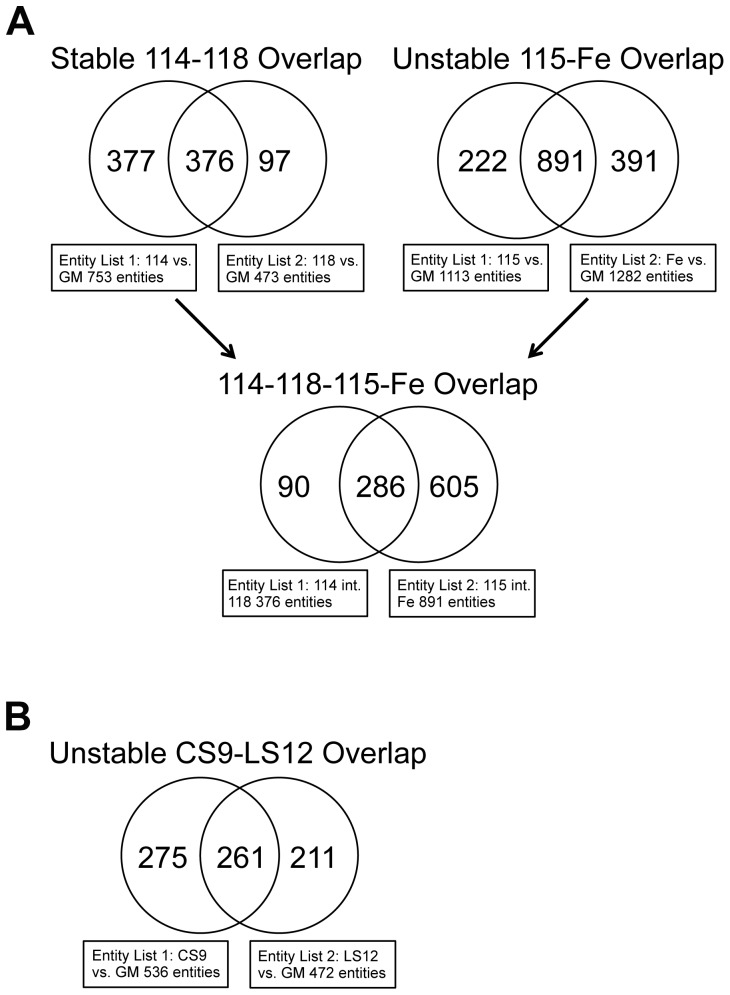
Overlap in gene expression profiles. The differentially regulated genes represented in each Venn diagram were identified with multiple testing and false discovery rate statistics at *P*<0.05. Three replicate arrays were performed and the significance threshold was set at *P*<0.05.

To determine whether changes in gene expression were linked to genomic instability mRNA arrays were performed. Interestingly, rather than correlation based on genome stability status, the array groupings most closely correlated with the repeat element DNA methylation profiles. Qualitatively, evaluation of simple heat maps from these expression arrays demonstrated clear trends such that the CS9 and LS12 clones were most similar to each other while 115 and Fe5.0–8 unstable clones had similar expression profiles to each other and to the stable clones 114 and 118 (Figure 4). The 115 and Fe5.0–8 unstable clones and the 114 and 118 stable clones had 376 and 891 overlapping mRNA, respectively, and when these four clones were compared together, 286 common mRNA were identified (Figure 5A). The CS9 and LS12 clones had 261 mRNA in common (Figure 5B).

Particularly for the genes differentially expressed by the 115 and Fe5.0–8 and 114 and 118 clones, Kyoto Encyclopedia of Genes and Genomes (KEGG) analyses identified significant enrichment of genes involved in signaling pathways affecting neurodegenerative diseases including Parkinson's, Alzheimer's and Huntington's diseases (Table 2). The Alzheimer's disease pathway was also enriched for the CS9 and LS12 unstable clones. While many of these pathways may seem unrelated to the study of radiation induced genomic instability these pathways are dominated by genes involved in redox reactions and the mitochondria. The themes of mitochondrial (dys)function and oxidative stress are commonly invoked in genomic instability. Reinforcing this impression, for the 115 and Fe5.0–8 and 114 and 118 clones genes involved in glutathione metabolism were enriched, and for the 115 and Fe5.0–8 cells tricarboxylic acid cycle (TCA) pathway genes were enriched. The gene names in bold type highlight genes related to mitochondrial function, oxidative stress and cellular metabolism.

**Table 2:** Canonical pathways predicted by KEGG analysis of mRNA levels.

115 int Fe5.0–8				
KEGG Pathway	mRNA	*P* Value	Genes	Fold
	Count			Enrichment
mmu03010:Ribosome	41	**1.61E**–**25**	RPL18, RPL17, RPL19, RPL13, RPL15, RPL35, RPL37,	7.16
			RPS27L, RPS2, RPS3, RPS26, RPS27, RPL32, RPL7,	
			RPS29, RPL6, RPS3A, RPL9, RPL34, RPL8, RPLP1,	
			RPL10, RPL7A, RPL12, RPS21, RPS23, RPL26,	
			RPL27, RPL24, RPS5, RPS8, RPS7, RPS18, RPS19,	
			RPL23, RPS16, RPL13A, RPS17, RPS13, RPL37A,	
			RPS11	
mmu05012:Parkinson's	25	**2.29E**–**07**	**UQCRC2, NDUFB4, ATP5B, ATP5G2, ATP5G1,**	3.31
disease			**COX5A, UQCRFS1, COX5B, ATP5G3, NDUFS7,**	
			**COX6B1, ATP5H, COX7A2, SLC25A4, SLC25A5,**	
			**CYCS, COX8A, VDAC2**, UBE2L3, **NDUFA1, SDHA,**	
			SDHB, ATP5C1, COX6A1, ATP5A1	
mmu03050:Proteasome	15	**8.07E**–**07**	SHFM1, PSMA2, PSMA1, PSMB4, PSMF1, PSMB7,	4.89
			PSMA6, PSMB1, PSME1, PSMD12, PSME2, PSMA5,	
			PSMB3, PSMB2, POMP	
mmu00190:Oxidative	24	**1.77E**–**06**	**UQCRC2, NDUFB4, ATP5J2, COX7A2, ATP5B,**	3.06
phosphorylation			**ATP5G2, ATP5G1, UQCRFS1, COX5A, COX5B,**	
			**COX8A, NDUFA1, ATP5G3, PPA1, ATP6V1F, SDHA,**	
			**NDUFS7, SDHB, ATP5C1, COX6B1, COX6A1, ATP5L,**	
			**ATP5A1, ATP5H**	
mmu05016:Huntington's	29	**4.18E**–**06**	**UQCRC2, NDUFB4**, CLTA, **ATP5B**, TBP, **ATP5G2**,	2.58
disease			**ATP5G1**, CLTC, **UQCRFS1, COX5A, COX5B**,	
			**ATP5G3, NDUFS7, GPX1, COX6B1, ATP5H, COX7A2,**	
			**SLC25A4, SLC25A5, CYCS, COX8A, VDAC2,**	
			**NDUFA1**, DCTN1, SDHA, SDHB, **ATP5C1, COX6A1,**	
			**ATP5A1**	
mmu05010:Alzheimer's	27	**1.31E**–**05**	**UQCRC2, HSD17B10, NDUFB4, ATP5B, ATP5G2,**	2.54
disease			**ATP5G1, COX5A, UQCRFS1, COX5B, ATP5G3,**	
			**NDUFS7, APP, COX6B1, ATP5H, GAPDH, COX7A2,**	
			**CYCS, COX8A, NDUFA1, SDHA, SDHB, ATP2A2,**	
			ERN1**, ATP5C1, COX6A1, CALM3, ATP5A1, CALM2,**	
			**CALM1**	
mmu03040:Spliceosome	17	**6.12E**–**03**	SNRPA1, SNRPD3, 0610009D07RIK, SNRPB2,	2.10
			SNRPD2, DDX5, HNRNPA1, SART1, CTNNBL1,	
			SF3B2, PRPF19, DDX46, RBM8A, BAT1A, SNRPC,	
			THOC2, THOC1	
mmu04260:Cardiac	12	**7.70E**–**03**	**UQCRC2, COX7A2, ATP2A2, ATP1B3, COX8A,**	2.48
muscle contraction			**COX6B1, COX6A1, ATP1A1, UQCRFS1, COX5A,**	
			TPM1, **COX5B**	
mmu04114:Oocyte	14	**3.00E**–**02**	ANAPC5, CAMK2G, YWHAB, CDC23, ANAPC11,	1.90
meiosis			SKP1A, PTTG1, PPP1CC, YWHAE, IGF1R, PLK1,	
			YWHAQ, **CALM3**, FBXW11, **CALM2, CALM1**	
mmu00020:Citrate cycle	6	**4.32E**–**02**	**SDHA, SDHB, IDH3G, SUCLG1, MDH2, MDH1**	3.04
(TCA cycle)				
mmu00480:Glutathione	8	5.48E–02	MGST3, GSTM1, ODC1, GPX1, SRM, RRM1, GSTM6,	2.30
metabolism			GSTM5	
mmu03010:Ribosome	16	**5.84E**–**08**	RPL18, RPL19, RPS27L, RPS3, RPS7, RPS26, RPS19,	5.76
			RPS16, RPL32, RPL7, RPL6, RPL34, RPLP1, RPL10,	
			RPS13, KPNA2	
mmu03050:Proteasome	10	**1.13E**–**05**	PSMA2, PSMB4, PSMA1, PSMB7, PSMD12, PSMB1,	6.72
			PSMA5, PSMB3, PSMB2, SHFM1	
mmu05012:Parkinson's	15	**1.25E**–**05**	**UQCRC2, NDUFB4, SLC25A5, ATP5B, CYCS,**	4.09
disease			**COX5A**, UBE2L3, **VDAC2, ATP5G3, NDUFS7**,	
			**ATP5G1, ATP5C1, COX6B1, COX6A1, ATP5A1**	
mmu05016:Huntington's	16	**2.87E**–**04**	**UQCRC2, NDUFB4,** CLTA**, SLC25A5, ATP5B, CYCS,**	2.93
disease			**ATP5G1, COX5A, VDAC2, ATP5G3, NDUFS7, GPX1,**	
			**ATP5C1, COX6B1, COX6A1, ATP5A1**	
mmu00190:Oxidative	13	**3.38E**–**04**	**UQCRC2, NDUFB4, ATP5B, ATP5G1, COX5A,**	3.42
phosphorylation			**ATP5G3, ATP6V1F, NDUFS7, ATP5C1, COX6B1,**	
			**ATP5L, COX6A1, ATP5A1**	
mmu05010:Alzheimer's	14	**1.66E**–**03**	**UQCRC2, HSD17B10, NDUFB4, ATP5B, CYCS,**	2.71
disease			**ATP5G1, COX5A, ATP5G3, NDUFS7, ATP5C1,**	
			**COX6B1, COX6A1, ATP5A1, CALM2, CALM1**	
mmu03040:Spliceosome	11	**5.36E**–**03**	SNRPA1, SNRPD3, 0610009D07RIK, SNRPD2,	2.79
			THOC2, SNRNP27, HNRNPA1, SNRPE, HSPA8,	
			SNRPC, THOC1	
mmu05322:Systemic	8	**9.52E**–**03**	HIST1H2BC, ACTN4, SNRPD3, HIST1H3A, H2AFZ,	3.31
lupus erythematosus			ACTN1, H3F3A, CBX3	
mmu00480:Glutathione	6	**2.50E**–**02**	MGST3, ODC1, GPX1, RRM1, GSTM6, GSTM5	3.56
metabolism				
mmu04520:Adherens	7	**3.56E**–**02**	CDC42, ACTN4, NLK, RAC1, RHOA, ACTN1, ACP1	2.82
junction				
mmu04114:Oocyte	8	6.35E–02	CDK1, PPP2CB, YWHAQ, ANAPC10, ANAPC11,	2.24
meiosis			SKP1A, PPP1CC, **CALM2, CALM1**	

CS9 int LS12				
KEGG Pathway	mRNA	*P* Value	Genes	Fold
	Count			Enrichment
mmu04142:Lysosome	9	**5.40E**–**03**	ATP6V0C, LAMP1, CTSZ, CLTA, LAMP2, IGF2R,	3.29
			M6PR, ATP6V0B, GBA	
mmu05010:Alzheimer's	10	**1.08E**–**02**	**HSD17B10,** APP, ADAM10**, ATP2A2, NDUFB9,**	2.69
disease			**NDUFV2, CYCS, COX6B1, GAPDH, CALM2**	
mmu04612:Antigen	5	6.93E–02	PDIA3, TAP2, HSPA5, CALR, CANX	3.18
processing/presenting				
mmu04260:Cardiac	5	8.56E–02	**ATP2A2, ATP1B3, COX6B1, ATP1A1,** TPM2	2.96
muscle contraction				

**Bold type** highlights genes related to mitochondrial function, oxidative stress and cellular metabolism.

### miR Array Analyses

**Figure 6:**
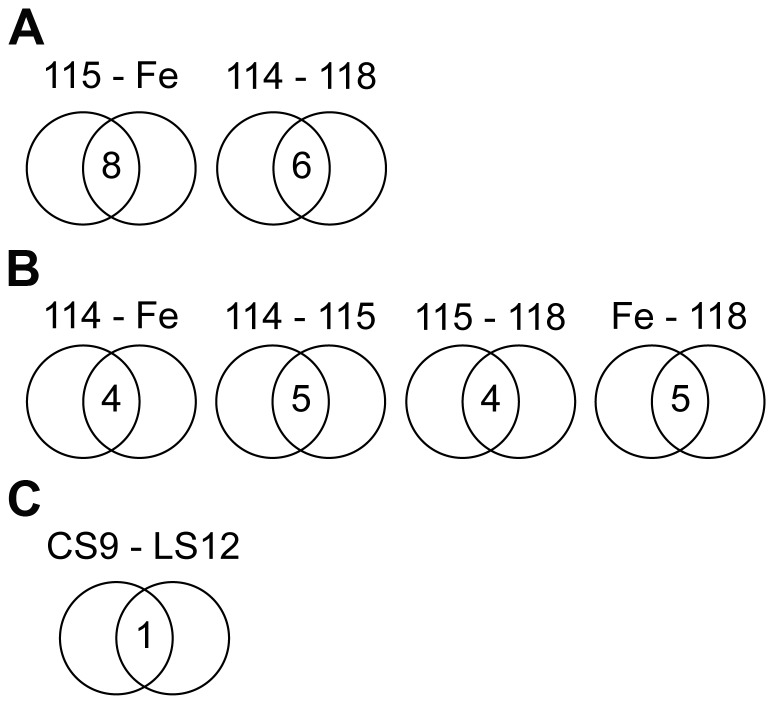
Overlap in miR expression profiles. Preliminary statistical analyses were performed on raw data normalized by the LOWESS method on the background-subtracted data. ANOVA were then performed to identify differences in miR expression. Two replicate arrays were performed, so the significance threshold was set at *P*<0.10.

To evaluate the possibility that miR epigenetically regulate the unstable phenotype, miR arrays were performed and analyzed based on defined criteria (*P*≤0.05, signal ≥100, fold change ≥1.5). These observations again correlated with the repeat element DNA methylation profiles, as well as the gene expression profiles. The highest levels of overlap occurred for the 115 and Fe5.0–8 unstable clones, followed by the stable 114 and 118 clones, with 8 and 6 miR in common, respectively (Figure 6A). The 8 miR found up regulated for the 115 and Fe5.0–8 grouping were all unique from the 6 miR identified for the 114 and 118 clones (Table 2). Additionally, if miR expression from any one of these four clones was compared to another of the four clones, 4 or 5 overlapping miR were identified, emphasizing the commonalities among the four clones (Figure 6B). The CS9 and LS12 unstable clones had 1 commonly expressed miR that was not common to any of the other four clones (Figure 6C).

To determine whether the miR that were differentially expressed might exert a functional epigenetic effect in these cells, the mRNA and miR arrays were merged and the predicted canonical pathways evaluated (Table 3). Those pathways for which statistical significance was obtained typically involve basic cellular processes including protein transport and metabolism, suggesting the possibility that these cells compensate for suboptimal basic cellular functions induced by the history of irradiation. For the 115 and Fe5.0–8 grouping, 7 of the 8 miR had anti-correlated mRNA levels for 96 genes (mmu-miR-325*, hsa-miR-1266, hsa-miR-1269, hsa-miR-1322, hsa-miR-27b*, hsa-miR-28-5p, hsa-miR-616; Table S2). For the 114 and 118 grouping 6 miR had anti-correlated mRNA levels for 50 genes (mmu-miR-805, hsa-miR-518c, hsa-miR-519c-3p, hsa-miR-520a-3p, hsa-miR-527, hsa-miR-606; Table S3). Although all 13 of the miR are unique, 6 of the anti-correlated mRNA are common to all four of these clones and are highlighted by bold font in Tables S2 and S3. In other cases, while not the same genes, the miR anti-correlated mRNA common to these four clones were from the same gene families.

**Table 3:** MiR overlap.

115 int Fe	115		Fe5.0–8	
miR Name	miR Log Ratio	*P* value	miR Log Ratio	*P* value
mmu-miR-325*	−0.58	0.07	−0.64	0.06
hsa-miR-27b*	1.21	0.09	1.56	0.08
hsa-miR-28-5p	−0.58	0.05	−0.95	0.03
hsa-miR-616	−0.53	0.09	−0.28	0.05
hsa-miR-1266	2.44	0.09	2.51	0.08
hsa-miR-1269	2.23	0.10	2.46	0.05
hsa-miR-1322	−0.95	0.05	−1.38	**0.04**
hsa-miR-1469	2.01	0.10	2.63	**0.04**

114 int 118	114		118	
miR Name	miR Log Ratio	*P* value	miR Log Ratio	*P* value
mmu-miR-805	2.71	0.06	2.57	0.07
hsa-miR-518c	−1.17	0.07	−1.65	0.06
hsa-miR-519c-3p	−0.79	0.06	−2.09	0.09
hsa-miR-520a-3p	−1.28	0.07	−1.6	0.06
hsa-miR-572	0.66	0.09	1.08	0.09
hsa-miR-606	−1.46	**0.05**	−1.64	**0.04**

The 6 anti-correlated mRNA that were common to the 115, Fe5.0–8, 114 and 118 clones are *solute carrier family 35 (UDP-galactose transporter) member A2, clatherin light chain alpha, chromodomain helicase DNA binding protein 2, slingshot homolog 2, caldesmon binding protein 1, and poly(A) polymerase alpha (SLC35a2, CLTA, Chd2, Ssh2, Cald1, Papola,* respectively). *SSH2, Cald1, Chd2* and *PAPOLA* are all up regulated for all four clones, *Slc35a2* is down-regulated in all four clones, and *CTLA* is up-regulated for 115 and Fe5.0-8 and down-regulated for 114–118.

## Discussion

We have tested the hypothesis that clones exhibiting radiation induced genomic instability have unique genetic and epigenetic changes that are different from irradiated, but chromosomally stable cells and as compared to the parental cell line. Further, we hypothesized that these changes play a mechanistic role in the unstable phenotype. These changes were evaluated in two stable clones that had been exposed to 10 Gy of low LET X-rays (clones 114, 118), three unstable clones that had been exposed to 10 Gy of X-rays (clones CS9, LS12, 115) and one clone that had been exposed to 5 Gy of high LET Fe ions (clone Fe5.0–8). Surprisingly, while significant genetic and epigenetic differences were observed in our unstable cells, similar changes were also frequently seen in the chromosomally stable cells. These findings suggest that the observed alterations may be representative of persistent effects of irradiation on that surviving progenitor cell rather than a signature of a mechanism driving genomic instability. Alternatively, while clones 114 and 118 do not exhibit chromosomal instability as measured by FISH analysis, they may have some other form of genomic instability for which they have not been evaluated such as expanded simple tandem repeat (ESTR) instability [18].

In this study the observed spectrum of changes in our stable and unstable cells included possible deletion events, and altered DNA methylation, mRNA and miR levels. These changes suggest that some cells might compensate for this history of irradiation better than others or at least in different ways. The changes also suggest that there are likely numerous mechanisms or pathways employed to compensate for deficiencies and to maintain cellular homeostasis. Although, it has been suggested previously that *NFκB* plays a role in the unstable phenotype observed in these cell lines [34], our data do not support this hypothesis given that we observed both increases and decreases in mRNA levels for CHO and human *NFκB* genes in the stable and unstable clones with no consistent trends. Future studies will require follow up work to determine whether the changes in mRNA level are reflective of NFκB protein levels. Similarly, while changes in global and repeat element DNA methylation were observed, there was a lack of consensus among the four unstable clones. However, the common LINE-1 DNA hypermethylation observed for the CS9 and LS12 clones and the common Alu element DNA hypomethylation observed for the 115 and Fe5.0–8 emerged as the first evidence of a compelling story. While it is possible that our data are merely correlative, the persistence of these unique groupings among the clones is also quite clear at the level of mRNA and miR expression.

We have only evaluated one clonally expanded single cell survivor of high LET irradiation so no conclusions can be drawn, but some observations might be made. The Fe5.0–8 cell line was exposed to 5 Gy of Fe ions as compared to 10 Gy of X-rays for the other clones. This might lead one to expect differences in phenotype based on radiation quality. However, the types of epigenetic and genetic changes that we observed for the Fe5.0–8 clone are not different from the low LET irradiated clones in any obvious way. High LET radiation exposure causes a different spectrum of DNA damage and generally has a higher relative biological effectiveness (RBE) for cell killing than low LET irradiation. In the original study that generated the Fe5.0–8 cell line Limoli and colleagues reported that the RBE of Fe ions for cell killing was 2, while the RBE for inducing chromosomal instability was only 1.3 [38]. For this reason, the Fe5.0–8 cell line may have differences in endogenous DNA damage without differences in other aspects of their phenotype or genomic instability.

The results of this study also reinforce the role of oxidative stress and mitochondrial function in the radiation response and genomic instability. Oxidative stress has been clearly shown to persist in these chromosomally unstable cell lines [8], [9], [39]–[41]. In the current paper KEGG pathway analyses were performed based on the three different clone groups, CS9-LS12, 115-Fe5.0-8, and 114–118. These analyses demonstrate significant enrichment of pathways related to oxidative stress, mitochondria and cellular metabolism. While the CS9 and LS12 clones showed the fewest common mRNA changes as a pair, these are probably the two best characterized clones with respect to documentation of persistent oxidative stress and mitochondrial dysfunction [8], [9], [11], [39], [41]. When evaluated independently in a separate proteomics study the LS12 clone had significant enrichment for electron transport chain and cellular redox homeostasis pathway proteins, and some of those genes were shown to be under epigenetic regulation by miR [11]. If the enrichment of the mRNA for similar mitochondrial genes, and oxidative stress and cellular metabolism pathways identified in our current study also translate into altered protein levels and activities, then they are likely to represent deficiencies as well as compensatory strategies. These changes may allow cells to survive under oxidative stress in some cases and/or compensate for suboptimal mitochondrial function in others.

While protein levels or enzyme activities were not evaluated in the current study we can also postulate some potential compensatory or detrimental effects of the epigenetically regulated changes in gene expression for the six miR anti-correlated mRNA that were common to the 115, Fe5.0–8, 114 and 118 clones. There are roles for Ssh2, caldesmon, clatherins, SLC35a2, poly(A) polymerase and Chd2 in everything from actin regulation, endocytosis, galactose transport, pre-mRNA poly adenylation to chromatin structure, DNA damage responses and genomic instability [42]–[50]. Altogether, the changes in mRNA levels for these six genes suggest possible defects in cellular processes as well as potential compensatory strategies that may have been induced each different clone by the irradiation of each different parent cell.

Other studies characterizing these cell lines have not eliminated the possibility that the two clones that are cytogenetically stable exhibit other forms of genomic instability [18]. However, if no other form of instability is found, then our data might suggest that one subset of the epigenetic and genetic changes that we have observed may represent a sort of radiation signature, exhibited by the progeny of certain cells that have survived high dose exposure while another subset of the observed epigenetic and genetic changes may contribute to persistent radiation induced genomic instability or be a result of that genomic instability. Irrespective, it is clear that genomic instability manifests in many different ways and a variety of mechanisms both drive this effect and permit unstable cells to persistent and proliferate.

## Supporting Information

Table S1 **PCR and qRT-PCR primer pairs. LS, Low Stringency; HS High Stringency.**
(DOCX)

Table S2 **miR with anti-correlated mRNA for 115-Fe5.**
(DOCX)

Table S3 **miR with anti-correlated mRNA for 114-118.**
(DOCX)

Workbook S1 **MiR identified as differentially regulated for each clone as compared to the parental GM10115 cell line.**
(XLSX)

Workbook S2 **mRNA identified as differentially regulated for each clone as compared to the parental GM10115 cell line.**
(XLSX)
