# Supplementary material for: Genetic and Epigenetic Changes in Chromosomally Stable and Unstable Progeny of Irradiated Cells
Source: PLoS One. 2014 Sep 24;9(9):e107722. doi: 10.1371/journal.pone.0107722 (PMC4175465; doi:10.1371/journal.pone.0107722)
Supplement: Table S1 — PCR and qRT-PCR primer pairs. LS, Low Stringency; HS High Stringency. (DOCX) [file pone.0107722.s001.docx]

**Table S1.** PCR and qRT-PCR primer pairs.

|  |  |  |
| --- | --- | --- |
| **Locus / PCR** | **PCR Primer Sequence (5’-3’)** | **Anneal Temp. (°C)** |
|  |  |  |
|  |  |  |
| *NFκB* unmethylated | F: AAGTAGTTTAGATGTTAGTGAATG  R: AAACCCCCAAAAAACACCTAC CAAACCCAATAATCAAAAAACA | 48.9 |
| *NFκB* methylated | F: AAGTAGTTTAGATGTTAGTGAATG  R: CCAAAAAACGCCTACCGAACC CAATAATCGAAAAACG | 47.3 |
| *NFκB* genomic | F: AAGCAGCTCAGATGCCAGTGAATG  R: GGGCTCTGGCTTCCTAGCAGGGC | 66.0 |
| *NFκB* bisulfite sequencing | F: AAGTAGTTTAGATGTTAGTGAAGT  R; AAACTCTAACTTCCTAACAAAAC | 55.0 |
| *TSLC1* unmethylated | F: TTTATAGGGTTTTGTTTTTAGGTGG  R: CTCCCGAACTCCCGAACCCGAACG | 55.0 |
| *TSLC1* methylated | F: TTTATAGGGTTTTGTTTTTAGGTGG  R: CTCCCGAACTCCCGAACCCGAACG | 55.0 |
| *CDH1* unmethylated | F: GATTTGAATGAATGTGAATTAGGAAGTTTTTTTAGGTTGGTTTTG  R: TAACCAAACAAACACCAAACAAACACAAAACTCAAATACAATCA | 52.0 |
| *CDH1* methylated | F: ATTCGAACGAATGTGAATTAGGAAGTTTTTTTAGGTCGGTTTC  R: CCGAACAAACACGAAACTCGAATACGATCG | 48.6 |
| NFkB qRT-PCR | F: CACTGCTGTGCAGGATGAGAATGG  R: ACATAGTTGCAGATTTTGACCTG | 60.0 |
| NFKB CHO Nested  qRT-PCR | F: GCCAGAGAAGGGCACGACAAGATC  R: AGCTTGTAGAGCTGCAGCCTTGTG | 60.0 |
| NFkB Human Nested  qRT-PCR | F: GCCAAAGAAGGACATGATAAAGTT  R: AACTTATACAGCTGCAGCTTCACA | 55.0 |
| LINE | F: TTGAGTTGTGGTGGGTTTTATTTAG  R: TCATCTCACTAAAAAATACCAAACA | 50.0 |
| Alu | F: GATCTTTTTATTAAAAATATAAAAATTAGT  R: GATCCCAAACTAAAATACAATAA | 43.0 |
| MSAP-PCR (MLG2) | AACCCTCACCCTAACCCCGG | 40.0 (LS)  55.0 (HS) |
|  |  |  |

LS, Low Stringency; HS High Stringency
