# Supplementary material for: Genetic and Epigenetic Changes in Chromosomally Stable and Unstable Progeny of Irradiated Cells
Source: PLoS One. 2014 Sep 24;9(9):e107722. doi: 10.1371/journal.pone.0107722 (PMC4175465; doi:10.1371/journal.pone.0107722)
Supplement: Table S2 — miR with anti-correlated mRNA for 115-Fe5. (DOCX) [file pone.0107722.s002.docx]

| **Table S2.** miR with anti-correlated mRNA for 115-Fe5. | | | | | |  |  | |
| --- | --- | --- | --- | --- | --- | --- | --- | --- |
|  |  |  |  |  |  | | |  |
|  |  |  |  |  |  | | |  |
| **miR Name** | **115 miR Log Ratio** | **Fe5 miR Log Ratio** |  | **Gene Symbol** | **115 mRNA Log Ratio** | | | **Fe5 mRNA Log Ratio** |
|  |  |  |  |  |  | | |  |
|  |  |  |  |  |  | | |  |
| mmu-miR-325* | -0.58 | -0.64 |  | Srgap2 | 0.71 | | | 0.71 |
|  |  |  |  | Coro7 | 0.67 | | | 0.52 |
|  |  |  |  | Ubr5 | 0.26 | | | 0.51 |
| hsa-miR-1266 | 2.44 | 2.51 |  | Laptm4a | -1.23 | | | -1.80 |
|  |  |  |  | Eif4g3 | -1.20 | | | 0.54 |
|  |  |  |  | Spcs2 | -1.16 | | | -1.93 |
|  |  |  |  | Lyrm4 | -0.95 | | | -0.62 |
|  |  |  |  | Cops6 | -0.92 | | | -0.83 |
|  |  |  |  | Mmp14 | -0.87 | | | -1.34 |
|  |  |  |  | Aasdhppt | -0.82 | | | -0.77 |
|  |  |  |  | Capzb | -0.76 | | | -0.69 |
|  |  |  |  | Ergic2 | -0.70 | | | -1.38 |
|  |  |  |  | Hsbp1 | -0.68 | | | -1.85 |
|  |  |  |  | Arf4 | -0.63 | | | -1.08 |
|  |  |  |  | Aig1 | -0.62 | | | -0.70 |
|  |  |  |  | Rcc2 | -0.61 | | | 0.83 |
|  |  |  |  | Ciao1 | -0.59 | | | -0.66 |
|  |  |  |  | Rpl37 | -0.58 | | | -0.59 |
|  |  |  |  | Dnaja2 | -0.57 | | | -0.68 |
|  |  |  |  | Anapc11 | -0.54 | | | -0.52 |
|  |  |  |  | Matr3 | -0.53 | | | -0.73 |
|  |  |  |  | Hspa5 | -0.52 | | | -0.52 |
|  |  |  |  | Eif5a | -0.51 | | | -0.55 |
|  |  |  |  | Gabarap | -0.49 | | | -1.27 |
|  |  |  |  | Gstm5 | -0.47 | | | -0.66 |
|  |  |  |  | Tbc1d9b | -0.47 | | | -0.42 |
|  |  |  |  | Tceb2 | -0.45 | | | -0.55 |
|  |  |  |  | Pum1 | -0.42 | | | -0.60 |
|  |  |  |  | Kpna3 | -0.39 | | | -0.60 |
|  |  |  |  | Csrp1 | -0.39 | | | -0.55 |
|  |  |  |  | Cltc | -0.38 | | | -0.59 |
|  |  |  |  | Calm3 | -0.37 | | | -0.48 |
|  |  |  |  | Acp1 | -0.35 | | | -0.52 |
|  |  |  |  | Irf2bp2 | -0.34 | | | -1.05 |
|  |  |  |  | Pdrg1 | -0.31 | | | -0.48 |
|  |  |  |  | Cebpb | -0.29 | | | -0.33 |
|  |  |  |  | Bzw2 | -0.29 | | | -0.25 |
|  |  |  |  | Nudt21 | -0.28 | | | -0.48 |
|  |  |  |  | Aifm1 | -0.27 | | | -0.30 |
|  |  |  |  | Pomp | -0.23 | | | -0.42 |
|  |  |  |  | S100a11 | -0.23 | | | -0.21 |
|  |  |  |  | Serf2 | -0.20 | | | -0.53 |
| hsa-miR-1269 | 2.23 | 2.46 |  | Syngr1 | -0.86 | | | -0.65 |
|  |  |  |  | M6pr | -0.72 | | | -0.66 |
|  |  |  |  | Dag1 | -0.69 | | | -1.16 |
|  |  |  |  | Nptn | -0.64 | | | -1.49 |
|  |  |  |  | Tnpo1 | -0.56 | | | -0.73 |
|  |  |  |  | Psma1 | -0.54 | | | -0.63 |
|  |  |  |  | Pdgfra | -0.51 | | | -0.72 |
|  |  |  |  | Atrx | -0.47 | | | -1.02 |
|  |  |  |  | Rpl13 | -0.40 | | | -1.04 |
|  |  |  |  | Hn1 | -0.40 | | | -0.53 |
|  |  |  |  | Timp2 | -0.40 | | | -0.63 |
|  |  |  |  | Kpna3 | -0.39 | | | -0.60 |
|  |  |  |  | Pgrmc1 | -0.38 | | | -0.42 |
|  |  |  |  | Rnf41 | -0.36 | | | -0.58 |
|  |  |  |  | Irf2bp2 | -0.34 | | | -1.05 |
|  |  |  |  | Klf9 | -0.29 | | | -0.54 |
|  |  |  |  | Atp5g3 | -0.21 | | | -0.54 |
| hsa-miR-1322 | -0.95 | -1.38 |  | Lcor | 1.09 | | | 1.02 |
|  |  |  |  | Ddx46 | 0.86 | | | 0.87 |
|  |  |  |  | Tap2 | 0.72 | | | 0.73 |
|  |  |  |  | Slmap | 0.71 | | | 0.86 |
|  |  |  |  | **Ssh2** | **0.69** | | | **0.34** |
|  |  |  |  | Ctnnbl1 | 0.66 | | | 0.60 |
|  |  |  |  | Med13 | 0.63 | | | 0.63 |
|  |  |  |  | Ctcf | 0.62 | | | 0.73 |
|  |  |  |  | Taf2 | 0.53 | | | 0.40 |
|  |  |  |  | **Cald1** | **0.51** | | | **0.42** |
|  |  |  |  | Eif4g2 | 0.35 | | | 0.27 |
|  |  |  |  | Map4k4 | 0.30 | | | 0.68 |
| hsa-miR-27b* | 1.21 | 1.56 |  | Fkbp11 | -1.49 | | | -1.02 |
|  |  |  |  | Syngr1 | -0.86 | | | -0.65 |
|  |  |  |  | Cox6a1 | -0.82 | | | -1.17 |
|  |  |  |  | **Slc35a2** | **-0.38** | | | **-0.67** |
| hsa-miR-28-5p | -0.58 | -0.95 |  | Fdft1 | 1.28 | | | 1.01 |
|  |  |  |  | **Clta** | **1.07** | | | **-0.69** |
|  |  |  |  | Tlk1 | 0.85 | | | 1.02 |
|  |  |  |  | Ubn1 | 0.62 | | | 0.74 |
|  |  |  |  | **Cald1** | **0.51** | | | **0.42** |
|  |  |  |  | Chd3 | 0.34 | | | 0.54 |
|  |  |  |  | Pmpcb | 0.21 | | | 0.38 |
|  |  |  |  | **Papola** | **0.20** | | | **0.32** |
| hsa-miR-616 | -0.53 | -0.28 |  | **Chd2** | **1.08** | | | **1.43** |
|  |  |  |  | Atp2b1 | 0.96 | | | 0.81 |
|  |  |  |  | Smc5 | 0.85 | | | 0.62 |
|  |  |  |  | Tlk1 | 0.85 | | | 1.02 |
|  |  |  |  | Tap2 | 0.72 | | | 0.73 |
|  |  |  |  | Kif5b | 0.66 | | | 0.74 |
|  |  |  |  | Hoxc9 | 0.60 | | | 0.95 |
|  |  |  |  | Usp7 | 0.57 | | | 0.66 |
|  |  |  |  | Bicd2 | 0.57 | | | 0.80 |
|  |  |  |  | Rims2 | 0.51 | | | 0.86 |
|  |  |  |  | Mycbp2 | 0.49 | | | 0.40 |
|  |  |  |  | Cnot3 | 0.48 | | | 0.64 |
|  |  |  |  | Nktr | 0.45 | | | 0.44 |
|  |  |  |  | Ube2i | 0.42 | | | 0.41 |
|  |  |  |  | Fip1l1 | 0.40 | | | 0.46 |
|  |  |  |  | Becn1 | 0.35 | | | 0.31 |
|  |  |  |  | Nckipsd | 0.33 | | | 1.46 |
|  |  |  |  | Ints3 | 0.31 | | | 0.36 |
|  |  |  |  | Ptp4a1 | 0.31 | | | 0.48 |
|  |  |  |  | Map4k4 | 0.30 | | | 0.68 |
|  |  |  |  | **Papola** | **0.20** | | | **0.32** |
|  |  |  |  |  |  | | |  |
|  |  |  |  |  |  | | |  |
