# Supplementary material for: Genetic and Epigenetic Changes in Chromosomally Stable and Unstable Progeny of Irradiated Cells
Source: PLoS One. 2014 Sep 24;9(9):e107722. doi: 10.1371/journal.pone.0107722 (PMC4175465; doi:10.1371/journal.pone.0107722)
Supplement: Table S3 — miR with anti-correlated mRNA for 114-118. (DOCX) [file pone.0107722.s003.docx]

| **Table S3.** miR with anti-correlated mRNA for 114-118. | | | | |  |
| --- | --- | --- | --- | --- | --- |
|  |  |  |  |  |  |
|  |  |  |  |  |  |
| **miR Name** | **114 miR Log Ratio** | **118 miR Log Ratio** | **Gene Symbol** | **114 mRNA Log Ratio** | **118 mRNA Log Ratio** |
|  |  |  |  |  |  |
|  |  |  |  |  |  |
| mmu-miR-805 | 2.71 | 2.57 | Kpna2 | -1.04 | -0.61 |
|  |  |  | Slc39a10 | -0.92 | -0.58 |
|  |  |  | Tyms | -0.92 | -0.72 |
|  |  |  | Psmb7 | -0.84 | -0.58 |
|  |  |  | Rpl7 | -0.76 | -0.48 |
|  |  |  | Tm9sf3 | -0.75 | -0.33 |
|  |  |  | Slc17a6 | -0.71 | -0.76 |
|  |  |  | Rab11a | -0.66 | -0.38 |
|  |  |  | Sri | -0.63 | -0.38 |
|  |  |  | Psmb1 | -0.62 | -0.48 |
|  |  |  | Hnrnpf | -0.60 | -0.33 |
|  |  |  | Atp5b | -0.58 | -0.58 |
|  |  |  | Fxr1 | -0.56 | -0.39 |
|  |  |  | Cbx3 | -0.55 | -0.32 |
|  |  |  | Eif3j | -0.52 | -0.52 |
|  |  |  | Atp13a3 | -0.51 | -0.44 |
|  |  |  | Cdc42 | -0.50 | -0.40 |
|  |  |  | Etfa | -0.50 | -0.35 |
|  |  |  | Pten | -0.48 | -0.56 |
|  |  |  | Rps27l | -0.47 | -0.53 |
|  |  |  | Slc25a5 | -0.43 | -0.38 |
|  |  |  | **Slc35a2** | **-0.40** | **-0.35** |
|  |  |  | Tubb2c | -0.37 | -0.40 |
|  |  |  | Psmb2 | -0.37 | -0.35 |
|  |  |  | Prrx1 | -0.35 | -0.56 |
|  |  |  | Ndufb4 | -0.35 | -0.26 |
|  |  |  | Uqcrc2 | -0.33 | -0.26 |
|  |  |  | **Clta** | **-0.32** | **-0.28** |
|  |  |  | Gch1 | -0.31 | -0.26 |
|  |  |  | Psmd12 | -0.30 | -0.29 |
|  |  |  | Cops4 | -0.30 | -0.30 |
|  |  |  | Set | -0.30 | -0.32 |
|  |  |  | Dbi | -0.30 | -0.27 |
|  |  |  | Timm23 | -0.27 | -0.27 |
|  |  |  | Hsd17b10 | -0.24 | -0.26 |
| hsa-miR-518c | -1.17 | -1.65 | Son | 0.36 | 0.33 |
| hsa-miR-519c-3p | -0.79 | -2.09 | Ogt | 1.36 | 0.67 |
|  |  |  | **Chd2** | **0.71** | **0.48** |
|  |  |  | **Ssh2** | **0.60** | **0.48** |
|  |  |  | **Cald1** | **0.53** | **0.23** |
|  |  |  | Gtpbp2 | 0.53 | 0.26 |
|  |  |  | Tnrc18 | 0.52 | 0.38 |
|  |  |  | Ints6 | 0.47 | 0.24 |
|  |  |  | Brd4 | 0.25 | 0.22 |
|  |  |  | **Papola** | **0.21** | **0.26** |
| hsa-miR-520a-3p | -1.28 | -1.6 | Tnrc18 | 0.52 | 0.38 |
|  |  |  | Ints6 | 0.47 | 0.24 |
|  |  |  | **Papola** | **0.21** | **0.26** |
| hsa-miR-572 | 0.66 | 1.08 | Ncam1 | -3.74 | -3.32 |
|  |  |  | Ran | -1.05 | -0.50 |
|  |  |  | Thbs1 | -0.50 | -0.47 |
| hsa-miR-606 | -1.46 | -1.64 | Bptf | 0.26 | 0.31 |
|  |  |  |  |  |  |
|  |  |  |  |  |  |
